# Anti-Hepatocellular-Cancer Activity Exerted by β-Sitosterol and β-Sitosterol-Glucoside from *Indigofera zollingeriana* Miq

**DOI:** 10.3390/molecules25133021

**Published:** 2020-07-02

**Authors:** Tuong Kha Vo, Qui Thanh Hoai Ta, Quang Truyen Chu, Thuy Trang Nguyen, Van Giau Vo

**Affiliations:** 1 Vietnam Sports Hospital, Ministry of Culture, Sports and Tourism, Hanoi 100000, Vietnam; vt.kha@tdtt.gov.vn; 2 Institute of Research and Development, Duy Tan University, Danang 550000, Vietnam; tathoaiqui@duytan.edu.vn; 3 Institute of Natural Products Chemistry, Vietnam Academy of Science and Technology, Hanoi 100000, Vietnam; quangtruyen69@gmail.com; 4 Faculty of Pharmacy, Ho Chi Minh City University of Technology (HUTECH), Ho Chi Minh City 700000, Vietnam; nt.trang85@hutech.edu.vn; 5 Bionanotechnology Research Group, Ton Duc Thang University, Ho Chi Minh City 700000, Vietnam; 6 Faculty of Pharmacy, Ton Duc Thang University, Ho Chi Minh City 700000, Vietnam

**Keywords:** *Indigofera zollingeriana* Miq, β-sitosterol, β-sitosterol-glucoside, anticancer, HepG2, Huh7

## Abstract

*Indigofera zollingeriana* Miq (*I.* *zollingeriana*) is a widely grown tree in Vietnam. It is used to cure various illnesses. The purpose of this study was to investigate the chemical constituents of an *I. zollingeriana* extract and test its anticancer activity on hepatocellular cells (Huh7 and HepG2). The experimental results of the analysis of the bioactive compounds revealed that β-sitosterol (β-S) and β-sitosterol-glucoside (β-SG) were the main ingredients of the *I.* *zollingeriana* extract. Regarding anticancer activity, the β-S and β-SG of *I. zollingeriana* were found to exhibit cytotoxic effects against HepG2 and Huh7 cells, but not against normal human primary fibroblasts. The β-S was able to inhibit the proliferation of HepG2 and Huh7 cells in a dose-dependent manner with half-maximal inhibitory concentration (IC_50_) values of 6.85 ± 0.61 µg/mL and 8.71 ± 0.21 µg/mL, respectively (*p* < 0.01), whereas the β-SG IC_50_ values were 4.64 ± 0.48 µg/mL for HepG2 and 5.25 ± 0.14 µg/mL for Huh7 cells (*p* < 0.01). Remarkably, our study also indicated that β-S and β-SG exhibited cytotoxic activities via inducing apoptosis and activating caspase-3 and -9 in these cells. These findings demonstrated that β-S and β-SG from *I.* *zollingeriana* could potentially be developed into promising therapeutic agents to treat liver cancer.

## 1. Introduction

Liver cancer (LC) is one of the most frequent cancers and is the leading cause of cancer-associated death globally. In 2018, 0.84 million new cases and 0.78 million deaths were reported [1,2]. To improve the clinical efficacy of LC treatment, several current approaches, such as surgery, radiation therapy, or chemotherapy, are commonly used. Unfortunately, the current anti-cancer drugs may cause various adverse effects and could be associated with eventual drug resistance [3], preventing their clinical efficacy and wide application. On the other hand, natural antioxidants and many phytochemicals have recently been suggested as anti-cancer adjuvant therapies because of their anti-proliferative and pro-apoptotic properties [4,5]. Hence, the continuing search for anticancer agents/compounds from plants plays a importance role in investigating possible ways to produce safe treatments and to minimize the side effects caused by chemotherapy because natural herbal medicines own many advantages [6,7,8,9].

Traditional medicine is widely used in Asian countries because of the therapeutic activities, such as antioxidant, hepatoprotective, antidiabetic, anticancer, antimicrobial, antineurodegenerative, anti-inflammatory, antiangiogenic, and hypoglycemic effects in both animals and humans [10]. Over several decades, around 200 new chemical compounds have been approved to fight cancer, 50% of which have originally come from natural products and their modifications, which are shown to be safe and have many advantages [11,12]. Plant-based therapy is considered to be a promising source for the development of new chemical compounds with potential bioactivities [9,13,14,15,16]. Along this line, *Indigofera zollingeriana* Miq is used in traditional medicine for treating various diseases. Related species, such as *Indigofera tinctoria and Indigofera suffruticosa*, contain the well-known dye-stuff indigo, which is associated with the presence of flavone glycosides and their glucosides, including phenolic, glycerol derivatives, steroids, triterpenoids, and fatty acids [17]. Previous studies have suggested that extracts of these plants may exhibit various pharmacological properties, such as anti-inflammatory [18], antibacterial [19], antifungal [19], insecticidal, and anticancer activities [19], but the active ingredients and their possible mechanisms remain unclear.

In Vietnam, *I. zollingeriana* is a popular herbal tree and has been used to cure various conditions. Nevertheless, there is still limited information on the chemical constituents of *I. zollingeriana* Miq and its anticancer effect. The purpose of this study was to investigate the chemical constituents from *I. zollingeriana* extracts and discover their potential anti-hepatocellular-cancer mechanism on HepG2 and Huh7 using appropriate techniques.

## 2. Materials and Methods

### 2.1. Chemicals and Reagents

Camptothecin, DMSO (dimethyl sulfoxide), FBS (fetal bovine serum), HEPES, glucose, l-glutamine, phenol red, sodium bicarbonate, and streptomycin were purchased from Sigma-Aldrich (St. Louis, MO, USA). *N*-hexane, ethyl acetate, chloroform, silica gel, methanol, trichloroacetic acid, and TLC (thin layer chromatography) silica gel 60 F_254_ were obtained from Merck (Darmstadt, Germany). All other chemicals were of analytical grade.

### 2.2. Plant Material Extraction and Isolation

The *I. zollingeriana* was collected from Hai Duong province, north of Hanoi, Vietnam, in 2018. It was taxonomically authenticated by Prof. Vo, Hanoi Medical University. A specimen (voucher no. V571) was deposited there. The shade-dried and powdered aerial part of *I. zollingeriana* (5 kg) was macerated with n-hexane for 72 h. Then, the solvent was removed under low pressure to gain a crude n-hexane extract (500 g) after filtrating. This extract was fractionated using column chromatography (CC) over silica gel (20–100% ethyl acetate in *n*-hexane, 1:1 ethyl acetate in methanol, and 100% methanol) to give 10 fractions (A.1–10). The fraction A.7 (35.5 g) was fractionated using CC on a silica gel (3:7 ethyl acetate in *n*-hexane) to give eight fractions (Fr.A.7.1–8). The CC of fraction A.7.4 (5.5 g) on a silica gel (3:7 ethyl acetate in *n*-hexane) gave seven fractions (Fr.A7.4.1–7). Repeated CC of fraction A7.4.1.4 on a silica gel (80% chloroform in *n*-hexane) produced compound InE (105 mg). The fraction A7.6 (1.7 g) was washed using cold methanol to give compound InH (107 mg). Subsequently, the isolated compounds underwent structural elucidation using MS, ^1^H-NMR, and ^13^C-NMR spectroscopy (Supporting Information, Figures S1–S6).

### 2.3. Free Radical Scavenging Activity Assay

A 2,2,1-diphenyl-1-picrylhydrazyl (DPPH) radical scavenging assay was used to measure the antioxidant properties of compounds, as previously described [20]. Briefly, 50 μL of sample solutions were dissolved with a 2 mL DPPH• radical methanol solution ranging from 6.10 to 5 M concentrations. Then, the absorbance was measured at 515 nm after 16 min. The percentage of DPPH• radical inhibition was determined using the following equation: % Inhibition = [(A_C(0)_ − A_S(t)_)/A_C(0)_)] × 100, where A_C(0)_ and A_S(t)_ are the absorbance of the control (t = 0 min) and samples A_S(t)_ (t = 16 min). The 50%inhibition (IC_50_) was estimated based on a graph against the sample concentration, where ascorbic acid was used as a standard.

### 2.4. ABTS Radical Scavenging Assay

The ABTS (2,2′-azino-bis(3-ethylbenzothiazoline-6-sulfonic acid) radical scavenging activity of the samples was determined as previously described [21]. Briefly, ABTS was dissolved in distilled wate to a 7 mM concentration. The ABTS radical cation (ABTS+) was generated by reacting an ABTS stock solution with 2.45 mM potassium persulfate. To determinate the antioxidant capacity of the samples, the ABTS solution was diluted in ethanol and stabilized at 30 °C. A 1.0 mL of diluted ABTS solution was mixed with a 10 mL sample and the absorbance was then measured at 593 nm against a blank. The 50% inhibition (IC_50_) was estimated based on a graph against the sample concentration, where ascorbic acid was used as a standard.

### 2.5. Cell Culture

The HepG2, Huh7, and primary fibroblast (PF) cell lines were obtained from the Korea Cell Line Bank (Seoul, Korea). The HepG2 and PF cells were initially cultured in Minimum Essential Medium Eagle (EME), 10% fetal calf serum, and 1% antibiotic-antimycotic. The HepG2 and PF cells were then converted to Dulbecco’s Modified Eagle Medium (DMEM) supplemented with 10% fetal bovine serum (FBS), 2 mM l-glutamine, and 1% penicillin/streptomycin. These cell lines were cultured at 37 °C under a humidified atmosphere of 5% CO_2_.

### 2.6. Cell Viability Assay

The cell viability assay was performed as previously described [9]. Briefly, the HepG2 and Huh7 were plated at 5 × 10^4^ cells/well (1.0 × 10^4^ cells/well for the PF cells) in a 96-well plate, followed by treatment with β-S, β-SG, or camptothecin at various concentrations of 40, 20, 10, 5, 2.5, 1.25 μg/mL after 24 h of incubation. After 48 h of further incubation, the treated cells were washed with PBS twice and a 100 μL fresh medium was added into each well. Afterward, a 100 μL of CellTiter-Glo^®^ Luminescent reagent (Promega, Madison, WI, USA) was added to each well to measure the cell viability with a luminescence signal using a microplate reader (Perkin Elmer, Victor X5, Victor X5, Norwalk, CT, USA). The cell viability percentage was calculated by comparing the absorbance values between treated and untreated cells. The experiments were repeated at least three times for the statistical analysis. To further evaluate the morphology of the cells, optical microscopy was also used during the experiment.

### 2.7. Analysis of DNA Fragmentation

The induction of apoptosis in HepG2 and Huh7 cells was evaluated using DNA fragmentation following treatment with β-S or β-SG at their IC_50_ doses after a 24 h exposure. Then, total DNA isolation was carried out using a DNA purification kit (Thermo Fisher Scientific, Santa Clara, CA, USA). After quantification, 2 μg of each DNA sample was applied to electrophoresis on a 1.5% agarose gel, and the gel was photographed under ultraviolet illumination after staining with ethidium bromide (10 μg/mL).

### 2.8. Cell Apoptosis Assay

The HepG2 or Huh7 cell apoptosis was further evaluated using an Annexin V-FITC/Propidium iodide (PI) binding assay according to the manufacturer’s protocol (Thermo Fisher Scientific, CA, USA). Briefly, 1 × 10^6^ cells were seeded in 6-well plates for 24 h before treatment with β-S or β-SG at their IC_50_ and 2 × IC_50_ concentrations for 24 h. The harvested cells were resuspended in a 1× binding buffer (0.1 M HEPES, 0.1 M NaOH at pH 7.4, 1.4 M NaCl, 25 mM CaCl_2_). The suspensions were then mixed with 5 μL of Annexin V-FITC, PI, and 400 μL of a 1× binding buffer. Then, the fluorescence of the samples was analyzed using flow cytometry (BD, Biosciences, San Jose, CA, USA). The FlowJo vX.0.7 (Tree Star, Inc., Ashland, OR, USA) was used to analyze the flow cytometry data.

### 2.9. Fluorescent Assays for Measuring Caspases Activity

Caspase-3, -8, and -9 activities were tested using the activity assay kit (Abcam, Cambridge, UK) according to the manufacturer’s guidelines. Briefly, HepG2 and Huh7 were cultured in 96-well plates at a density of 2 × 10^4^ cells/well and treated with β-S or β-SG at their IC_50_ concentration for 24 h. A total of 100 μL of caspase reagent was added to each well. The fluorescence intensity of each well was measured at Ex/Em = 535/620 nm, Ex/Em = 490/525 nm, and Ex/Em = 370/450 nm for caspase-3, -8, and -9, respectively, using a plate-reading fluorescence reader (Perkin Elmer, Victor X5, Norwalk, CT, USA).

### 2.10. Western Blot Analysis

Following treatment with β-S or β-SG at their IC_50_ values after 24 h of exposure, the HepG2 and Huh7 cells were harvested and the total proteins were extracted using a radioimmunoprecipitation assay, followed by centrifugation at 16,000× *g* for 20 min at 4 °C. The total proteins were loaded onto a 12% SDS-PAGE, separated, and transferred onto a nitrocellulose membrane. Then, 5% bovine serum albumin was used to block the membranes, which were then probed with active/cleaved caspase-3, -8, and -9 at 4 °C overnight. After washing three times with a TBST buffer (Tris-buffered saline, 0.1% Tween 20), the corresponding HRP-conjugated secondary antibody was added and incubated for 1 h at room temperature. The blots were visualized using enhanced chemiluminesence detection and quantified via densitometry using Image J version 1.47 (Bethesda, MD, USA).

### 2.11. Statistical Analysis

The experiments were repeated at least three times for the statistical analysis. The data are expressed as mean ± standard deviation and analyzed using the Statistical Package for Social Sciences (SPSS) (version 20.0) software (SPSS Inc., Chicago, IL, USA). A *p*-value of less than 0.01 was considered to be statistically significant.

## 3. Results and Discussion

### 3.1. Antioxidant Activity Assessments of Isolated Compounds

**Figure 1:**
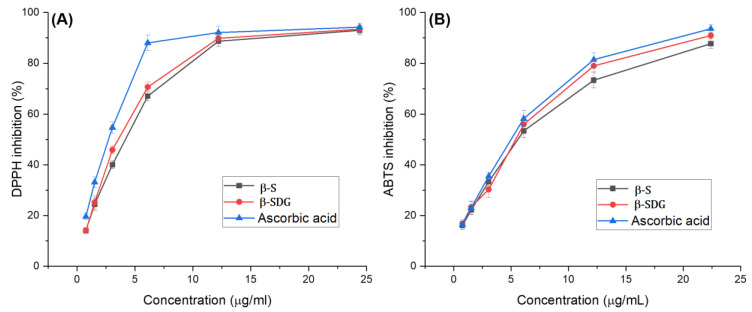
Antioxidant activity assessments of isolated compounds in terms of DPPH (**A**) and ABTS (**B**) radical scavenging activities.

Free radicals are highly reactive chemicals that have the potential to inhibit cancer cells, which is one type of antioxidant potential of plant extracts [22,23]. ABTS+ and DPPH assays are widely used to evaluate a compound’s antioxidant potential. The antioxidant activity of the compounds (InH and InE) and standard (ascorbic acid) are presented in Figure 1. As shown in Figure 1A, for the DPPH scavenging assay, the IC_50_ value of the InE compound was found to be 3.04 μg/mL, which was estimated to be 1.14-fold lower than for the ascorbic acid (IC_50_ = 2.65 μg/mL), followed by the InH compound with an IC_50_ value of 3.85 μg/mL (*p* < 0.01).

On the other hand, the ABTS+ assay is an additional important method for quantifying radical scavenging activity, which can provide comparable results to those obtained in the DPPH assay. As indicated in Figure 1B, the InH and InE compounds revealed excellent antioxidant properties in comparison to ascorbic acid, which is a well-known antioxidant. The IC_50_ value of the InE compound was 5.13 ± 0.16 µg/mL, followed by the InH compound with a value of 5.29 ± 0.32 µg/mL, which was compared to that of ascorbic acid (IC_50_ = 4.71 ± 0.25 µg/mL) (*p* < 0.01).

Ascorbic acid has been well documented as an excellent antioxidant and anticancer agent; as such it is widely used in the treatment and prevention of cancer [24,25]. In both the assays, the compounds exhibited good antioxidant/free-radical scavenger activity that was similar to the control antioxidant, ascorbic acid. Indeed, the correlation between antioxidant or scavenger activities in terms of the prevention of cancer has been well studied [26]. Some previous studies revealed that phenols and flavonoids have strong antioxidants that exhibit effective anticancer agents through anti-angiogenic and apoptosis activities [27,28]. In this study, both compounds are flavonoids that exhibit excellent antioxidant and cytotoxic effects.

### 3.2. Identification of β-S and β-SG

#### 3.2.1. Compound InH

**Figure 2:**
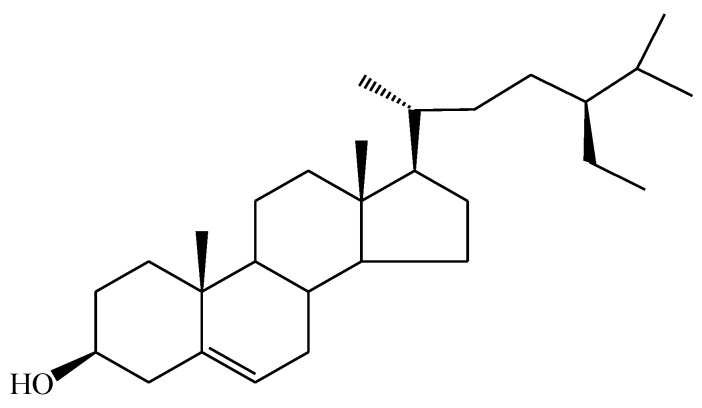
Chemical structure of β-sitosterol from the InH compound.

Compound InH was obtained as a light-yellow powder. 1H-NMR (CDCl3): δ (ppm) 0.68 (3H, *s*, 18-CH3), 0.93 (3H, *d*, *J* = 6.5 Hz, 21-CH3), 0.81 (3H, *d*, *J* = 6.8 Hz, 26-CH3), 0.84 (3H, *d*, *J* = 6.8 Hz, 27-CH3), 0.84 (3H, *t*, *J* = 7.4 Hz, 29-CH3), 1.00 (3H, *s*, 19-CH3), 3.53 (1H, *tt*, *J* = 4.8 Hz, 11.0 Hz, H-3), 5.35 (1H, *dd*, *J* = 5.2 Hz, H6). 13C NMR (100 Hz, CDCl3): δ 37.4 (C-1), 31.9 (C-2), 71.8 (C-3), 42.2 (C-4), 140.8 (C-5), 121.7 (C-6), 31.7 (C-7), 31.9 (C-8), 50.1 (C-9), 36.5 (C-10), 21.1 (C-11), 39.8 (C-12), 42.3 (C-13), 56.8 (C-14), 24.3 (C-15), 28.3 (C-16), 56.1 (C17), 11.9 (C-18), 19.4 (C-19), 36.2 (C-20), 18.8 (C-21), 34.0 (C-22), 26.1 (C-23), 45.8 (C-24), 29.2 (C-25), 19.8 (C-26), 19.0 (C-27), 23.1 (C28), 12.0 (C-29). These results were consistent with the published findings [29,30]. Figure 2 presents the chemical structure of the InH compound isolated from *I. zollingeriana* (see also Figures S1–S3 for the spectra). Thus, the target compound was identified as β-sitosterol (β-S).

#### 3.2.2. Compound InE

**Figure 3:**
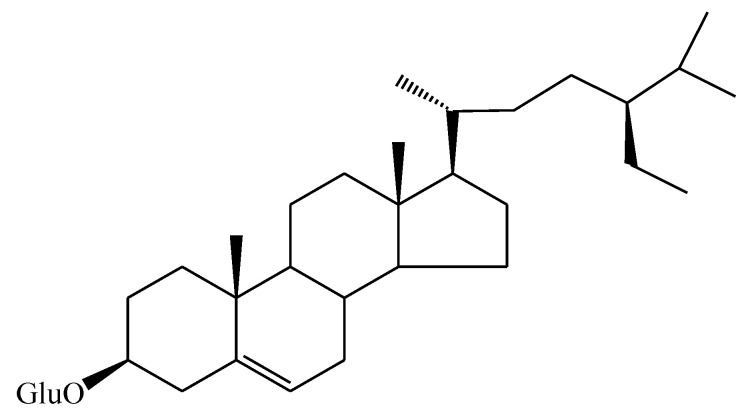
Chemical structure of β-sitosterol-glucoside from the InE compound.

Compound InE was obtained as a white powder. ^1^H-NMR (500 MHz, DMSO-*d*_6_), δ_H_ 5.31 (1H; *s*; H-6), δ_H_ 4.35 (1H; *d*; *J* = 9.4 Hz; H-1′), δ_H_ 3.54 (1H; *m*; H-3), δ_H_ 3.23 (1H; *s*; H-2′), δ_H_ 3.38 (1H; *m*; H-3′), δ_H_ 3.39 (1H; *m*; H-4′), δ_H_ 3.30 (1H; *s*; H-5′), δ_H_ 3.68 (1H; *d*; *J* = 10.3 Hz; H-6′), δ_H_ 3.70 (1H; *d*; *J* = 10.3 Hz; H-6′, δ_H_ 0.97 (3H; *s*; H-18), δ_H_ 0.65 (3H; *s*; H-19), δ_H_ 0.88 (3H; *d*; *J* = 6.3 Hz; H-21), δ_H_ 0.79 (3H; *d*; *J* = 6.6 Hz; H-26), δ_H_ 0.80 (3H; *d*; *J* = 7.0 Hz; H-27), δ_H_ 0.81 (3H; *t*; *J* = 6.6 Hz; H-29). ^13^C-NMR (125 MHz, DMSO-d_6_), δ(ppm): (δ_C_ 140.3; C-5); (δ_C_ 122.0; C-6); (δ_C_ 101.1; C-1‘); (δ_C_ 79.1; C-3),(δ_C_ 73.5; C-2‘), (δ_C_ 76.4; C-3‘), (δ_C_ 70.2; C-4′), (δ_C_ 75.7; C-5′), (δ_C_ 61.8; C-6′), *sp* 3 (δ_C_ 36.6; C-10), (δ_C_ 42.3; C-13), (δ_C_ 31.8; C-8), (δ_C_ 50.2; C-9), (δ_C_ 56.7; C-14), (δ_C_ 56.0; C-17), (δ_C_ 36.1; C-20), (δ_C_ 45.9; C-24), (δ_C_ 29.2; C-25), (δ_C_ 37.2; C-1), (δ_C_ 29.5; C-2), (δ_C_ 38.6; C-4), (δ_C_ 31.8; C-7), (δ_C_ 21.0; C-11), (δ_C_ 39.7; C-12), (δ_C_ 24.2; C-15), (δ_C_ 28.1; C-16), (δ_C_ 33.9; C-22), (δ_C_ 26.1; C-23), (δ_C_ 23.0; C-28), (δ_C_ 11.7; C-18), (δ_C_ 19.1; C-19), (δ_C_ 18.6; C-21), (δ_C_ 19.6; C-26), (δ_C_ 18.9; C-27), (δ_C_ 11.8; C-29). These findings were in line with the published findings [30,31]. Figure 3 reveals the chemical structure of the InE compound isolated from *I. zollingeriana* (see also Figures S4–S6 for the spectra). This compound was therefore established as being β-sitosterol-glucoside (β-SG).

β-S and β-SG have been widely found to be in different fruits and vegetables [31], and a variety of plants [32]. It has been suggested that both β-S and β-SG may exhibit antioxidant, antimicrobial, antidiabetic, anti-inflammatory, and anticancer activities without any harmful toxicity [33].

### 3.3. Cytotoxic Effect of β-S and β-SG on LC-Cell Proliferation and Normal Human Primary Fibroblasts

**Figure 4:**
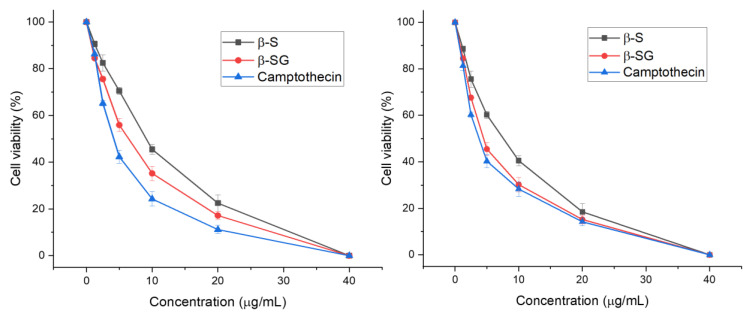
Cytotoxicity of the compounds and camptothecin on HepG2 (**A**) and Huh7 (**B**) cells at different concentrations.

**Figure 5:**
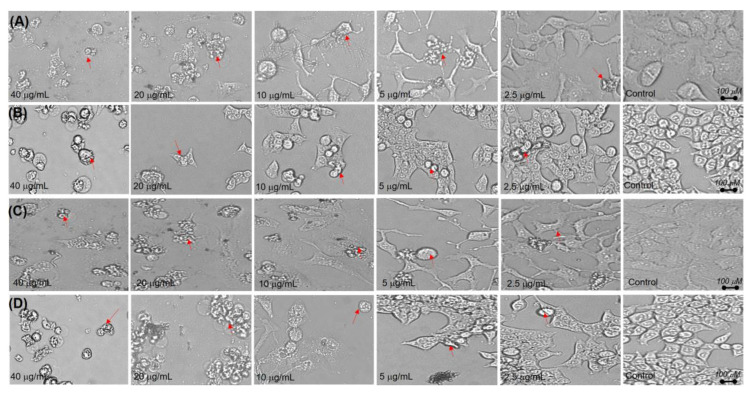
Morphological alterations of cells after 48 h caused by (**A**) β-S on HepG2 cells, (**B**) β-S on Huh7 cells, (**C**) β-SG on HepG2 cells, and (**D**) β-SG on Huh7 cells.

The survival of liver cancer cell lines (HepG2 and Huh7) was assessed using a cell viability assay following exposure to increasing doses (1.25, 2.5, 5, 10, 20, and 40 μg/mL) of β-S, β-SG, and camptothecin for 48 h (Figure 4). As shown in Figure 4, β-S showed half-maximal inhibitory concentration (IC_50_) values of 6.85 ± 0.61 µg/mL and 8.71 ± 0.21 µg/mL for HepG2 and Huh7 cell lines, respectively (*p* < 0.01), whereas the β-SG IC_50_ values were 4.64 ± 0.48 for HepG2 and 5.25 ± 0.14 µg/mL for Huh7 cells (*p* < 0.01). Similar results were observed for camptothecin with IC_50_ values of 3.8 ± 0.45 µg/mL for HepG2 and 4.25 ± 0.16 µg/mL for Huh7 (*p* < 0.01). According to the criteria of the National Cancer Institute and the Geran protocol, natural extracts with an IC_50_ value of ≤20 µg/mL considered highly cytotoxic [34,35,36]. Remarkably, no cytotoxic effect was observed on normal human primary fibroblasts (PFs) for the tested compounds within the test range (0–40 μg/mL) (Figure S7). In addition, the cell viability was more than 80%, even at compound concentrations of 40 µg/mL and morphological changes of PF cells were confirmed (Figure S8). Almost all the PF cells were still alive over the whole range of the concentrations, indicating that β-S and β-SG showed no obvious cytotoxicity toward normal cells. Hence, both β-S and β-SG compounds could be considered to have strong in vitro cytotoxic activities against HepG2 and Huh7 with IC_50_ values ≤10 μg/mL. Consistent with these cytotoxicity findings was a reduction of the cell populations and more cell shrinkage was observed when the β-S and β-SG doses increased compared to untreated cells (Figure 5), indicating that apoptosis was induced in HepG2 and Huh7 cells by the compounds. As a result of caspase activity, apoptotic cells begin to shrink and undergo plasma membrane changes that signal the macrophage response [37].

The available literature indicates that both β-S and β-SG exhibits excellent anticancer activity against several cancer cells like lung, breast, and liver [31,38]. Previously, β-S was isolated from different *Hibiscus* species and exhibited the most potent cytotoxic effect on HepG2 and MCF-7 cell lines (IC_50_: 14.4 and 11.1 μg/mL, respectively). In the present study, the β-S compound of *I. zollingeriana* was shown to provide approximately 2-fold more (*p* < 0.01) toxic effects. In other words, β-SG was previously isolated from sweet potato and possibly displayed potent anticancer activity with half-maximal inhibitory values of 30.82 μM and 49.76 μM for MCF7 and MDA-MB-231 cells, respectively [31]. The present results additionally confirmed that the β-SG provides strong anticancer effects against HepG2 and Huh7.

### 3.4. Analysis of DNA Fragmentation

**Figure 6:**
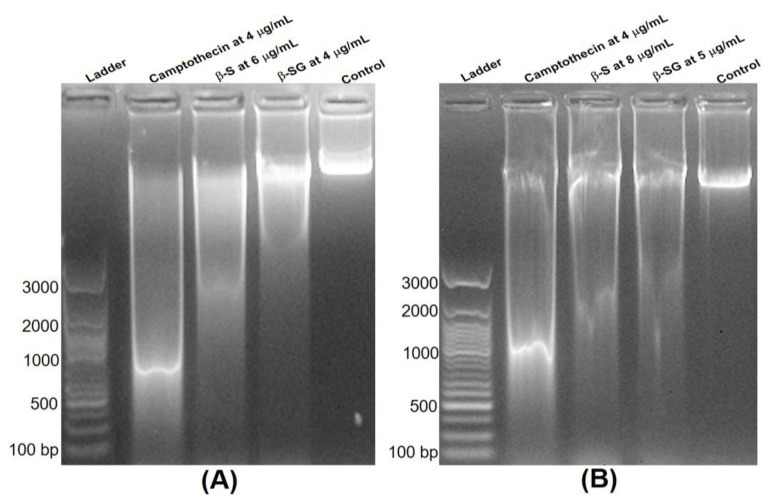
Apoptosis induced in HepG2 (**A**) and Huh7 (**B**) cells by β-S and β-SG at their IC_50_.

To confirm the apoptosis, DNA fragmentation was also tested via observation of the formation of the DNA ladder. As presented in Figure 6, no DNA fragmentation was detected in the control group, while a significant DNA fragmentation was obtained in the camptothecin treatment at 4 μg/mL. Interestingly, the treatment of β-S and β-SG at their IC_50_ doses after just 24 h of exposure also caused a substantial increase in DNA fragmentation, which indicated that the cytotoxic effect of the compounds might mediate through the induction of apoptosis. Some common chemotherapeutic drugs target cell cycle arrest and induce apoptosis to eradicate cancerous cells, such as doxorubicin [39], cisplatin [40], and tamoxifen [41], distinguishing between cells’ morphological changes, including membrane blebbing, cell shrinkage, and DNA fragmentation.

### 3.5. Quantification of Apoptotic Cell Death Using the Annexin V FITC Assay

Based on the morphology observation and DNA fragmentation, we investigated whether β-S and β-SG could induce apoptotic or necrotic cell death using FACS analysis. Double-staining using Annexin V-FITC and PI was applied to evaluate the apoptotic potential of the compounds. After the HepG2 and Huh7 cells were treated with different concentrations of β-S (6–16 μg/mL) and β-SG (4–10 μg/mL) for 24 h, the cells were stained with Annexin V-FITC and PI, which can show the percentage of viable, early, late, and dead cell populations, as presented in the lower-left, lower-right, upper-right, and upper-left quadrants of dot plots, respectively [42].

**Figure 7:**
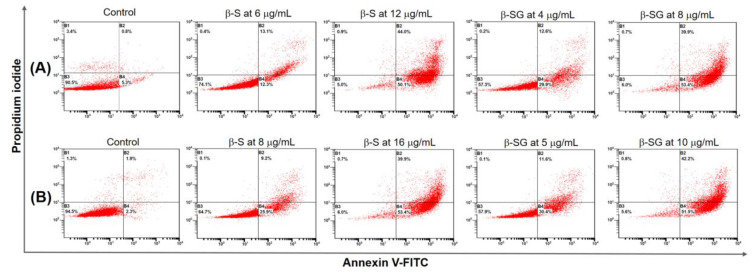
Quantification of apoptosis induced by β-S and β-SG in HepG2 (**A**) and Huh7 (**B**) cells evaluated using Annexin V-FITC/PI dual staining for 48 h and analyzed using fluorescence-activated cell sorting.

Compared to untreated cells, the β-S treatment at IC_50_ reduced the viable HepG2 and Huh7 cell populations to 24.2% and 30.2%, and increased the percentage of early apoptotic cells to 25.3% and 35.1%, respectively. Noticeably, the percent of viable and early apoptotic cells rapidly decreased, whereas the population of late apoptosis was significantly increased from 13.1% to 44% for HepG2 cells (Figure 7A) and 9.2% to 39.9% for Huh7 cells (Figure 7B) with increasing doses of β-S from IC_50_ to 2 × IC_50_, respectively. This result strongly indicated that apoptosis occurred in HepG2 and Huh7 cells due to the β-S in a dose-dependent manner. On the other hand, treatment with the β-SG at IC_50_ on HepG2 cells (Figure 7A) resulted in viable, early apoptotic, late apoptotic, and dead cell populations (57.3%, 0.2%, 12.6%, and 29.9%), while the respective values were 57.9%, 1.1%, 11.6%, and 30.4% for Huh7 cells (Figure 7B). Remarkably, at the 2 × IC_50_ dose level, the population of viable HepG2 (Figure 7A) and Huh7 (Figure 7B) cells were dramatically reduced to 51% and 49.8%, and that of early apoptotic cells was significantly increased to 27.3% and 30.6%, respectively. The current findings revealed that treatment with β-S and β-SG induced translocation of phospholipid phosphatidylserine on the surface of HepG2 and Huh7 in a dose-dependent manner, which might lead to DNA damage, and thereby cause an increase of apoptosis, as well as initiating cell death events [43]. Regarding the cytotoxicity effect, β-S and β-SG could lead to DNA damage and apoptosis, as seen in the number of cell death events, due to the number of contained ROS radicals [43,44,45].

### 3.6. β-S- and β-SG-Induced Apoptosis in Hepatocyte Carcinoma through Activating Both Caspase-3 and Caspase-9

**Figure 8:**
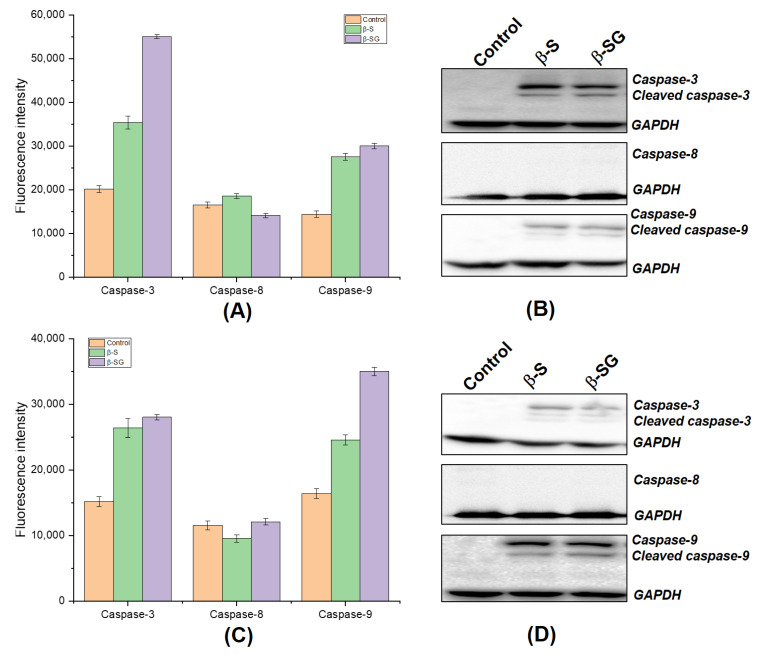
β-S and β-SG induced the death of liver cancer cells through caspase-dependent pathways. Measurement of the fluorescence intensity in response to caspase activities on HepG2 (**A**) and Huh7 (**B**) cells. Protein expression analysis of anti-active and its cleaved caspase-3, -8, and -9 on HepG2 (**C**) and Huh7 (**D**) cells. GAPDH was used as a loading control.

Two main signaling pathways initiate the apoptotic suicide program in mammalian cells, including the extrinsic (mediated by death receptors on the cell‘s surface) and the intrinsic (mediated by mitochondria) pathways [46,47], which involve a cascade activation of caspases. To determine the potential mechanism that might underlie the cytotoxic effects mediated by β-S and β-SG, the activities of caspase-3, -8, and -9 from HepG2 and Huh7 cells were measured using a fluorometric assay. As shown in Figure 8A–C, the exposure of cells to the β-S and β-SG of *I. zollingeriana* enhanced caspase-3 and -9 activations, while caspase-8 activity did not change significantly compared to the untreated groups. Indeed, a significant increase of caspase-3 activities was observed in both HepG2 and Huh7 cells treated with IC_50_ doses of the β-S compound by approximately 2.2 and 3.1-fold compared to untreated cells, respectively. Furthermore, there was nearly 2.2- and 1.8-fold increases of caspase-9 activities after the exposure of β-S at IC_50_ for HepG2 and Huh7 cells, respectively, when compared to the control. On the other hand, treatment with the β-SG at IC_50_ on HepG2 also resulted in a significant increase in caspase-3 activity (nearly 3-fold), while this figure was 1.9-fold for Huh7 cells. Similarly, β-SG was able to trigger caspase activity at similar levels in both cell lines. These results suggest that β-S and β-SG may inhibit the LC-cell growth in vitro via activated caspase-9 and caspase-3 pathways.

In addition, we further confirmed whether the increased caspase-3 and -9 activities in terms of fluorescence intensity measurements were due to the cleavage of procaspase-3 and procaspase-9 (inactive forms) into the corresponding active forms. Hence, the expression level of apoptosis-related active proteins caspase-3 and -9 were confirmed using a Western blot assay. The expression of active caspase-3 and -9 proteins were clearly observed due to the IC_50_ doses of β-S and β-SG treatments compared to the untreated group for HepG2 (Figure 8B) and Huh7 (Figure 8D) cells. A comparison with the fluorescence data showed that these results were consistent and strongly revealed that β-S and β-SG might induce apoptosis in human hepatocyte carcinoma by exerting caspase-3 and -9 activities.

**Figure 9:**
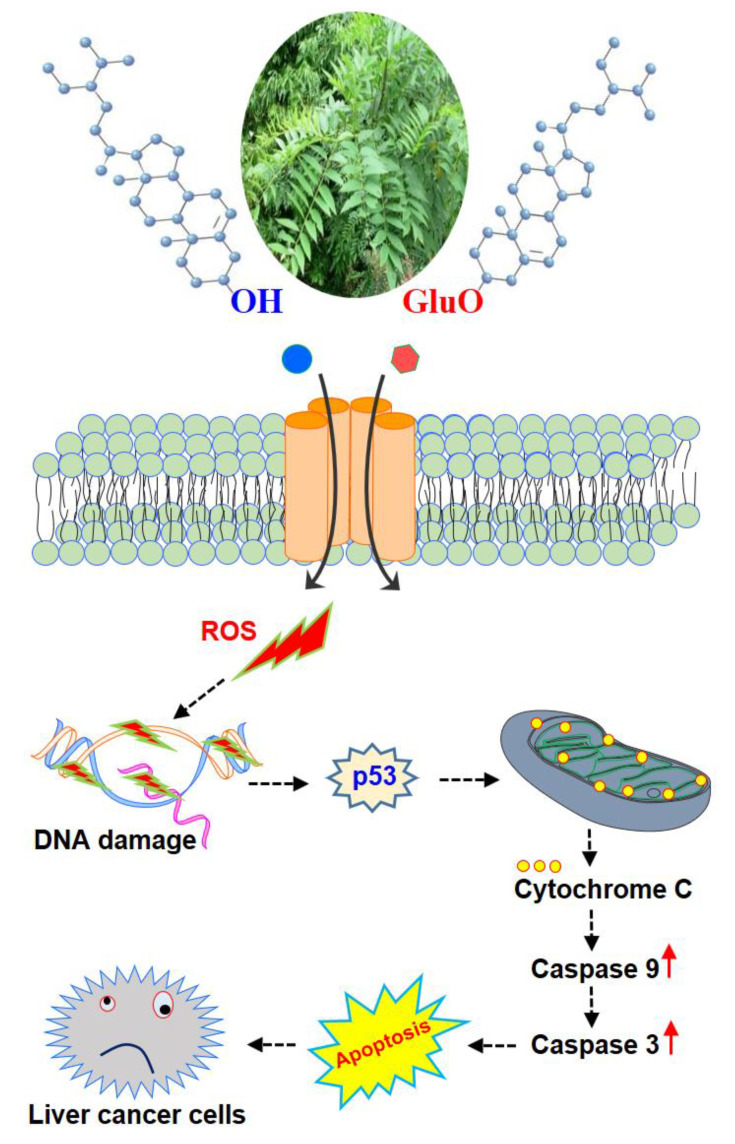
Schematic representation of the proposed signaling effect of β-S and β-SG on liver cancer cells. β-S and β-SG induce excessive ROS production through caspase-dependent pathways. ROS accumulation causes DNA damage, p53 activation, and triggers the mitochondrial-mediated apoptotic cell death in HepG2 and Huh7 cells.

Previously, the antiproliferative activity of β-S has been reported in breast cancer [48,49], colon cancer [50,51,52], and HeLa cells [53] due to caspase-induced apoptosis. In addition, β-SG was isolated from *Castanopsis indica* leaves and was demonstrated to induce apoptotic pathways through up-regulating caspase-9 and caspase-3 activities [54]. The current findings match with the previous studies since the β-S induced caspase-3 and 9 activations and apoptosis in cancer cells. Consistent with these previous reports, increased expression of cleaved caspase-3 and -9 in HepG2 and Huh7 cells were observed after treating with β-S and β-SG for 24 h. The interactions may result from new anticancer molecules in which antioxidants can also be enhanced to eliminate cancer cells through the apoptosis pathway, where antioxidants from medicinal plants have shown a great cytotoxic potential [55,56]. Taken together, the present study concluded that β-S and β-SG induced the ROS dependent apoptotic mode of cell death in HepG2 and Huh7 cells through the up-regulation of the caspase-3 and -9 signaling pathway (Figure 9). The results presented herein indicate that treatment with β-S and β-SG would be a potent strategy for targeted treatment of liver cancer. The search for new antioxidants containing a low toxicity profile is desirable and the *I. zollingeriana* demonstrated here may represent interesting targets for this purpose.

## 4. Conclusions

This study aimed to reveal the inhibitory effects of *I. zollingeriana* on the growth of human liver cancer cells. In addition, we demonstrated that β-S and β-SG exerted an anticancer effect in liver cancer cells by activating the caspase-3 and -9 signaling pathway associated with apoptosis cell death. These results suggest that *I. zollingeriana* is a promising source of useful natural products, and β-S and β-SG offer opportunities to develop novel anticancer drugs for treating cancers, including liver cancer.

## Supplementary Materials

The following are available online, Figure S1: ^13^C-NMR spectra of β-sitosterol. Figure S2: ^13^C-DEPT 90, 135, and CPD spectra of β-sitosterol. Figure S3: ^1^H-NMR of β-sitosterol-glucoside. Figure S4: ^1^H-NMR of β-sitosterol-glucoside. Figure S5: ^13^C-NMR of β-sitosterol-glucoside. Figure S6: ^13^C-DEPT 90, 135, and CPD spectra of β-sitosterol-glucoside. Figure S7: Cytotoxicity of the compounds on normal human primary fibroblast (PF) cell at different concentrations for 48 h. Figure S8: Morphological alterations of cells caused by β-S (A) and β-SG (B) on primary fibroblast cells at different concentrations for 48 h.
