# Supplementary material for: Anti-Hepatocellular-Cancer Activity Exerted by β-Sitosterol and β-Sitosterol-Glucoside from Indigofera zollingeriana Miq
Source: Molecules. 2020 Jul 2;25(13):3021. doi: 10.3390/molecules25133021 (PMC7411723; doi:10.3390/molecules25133021)
Supplement: Supplementary file 1 [file molecules-25-03021-s001.pdf]

*Supplementary Material*

# Anti-hepatocellular-cancer activity exerted by β-sitosterol and β-Sitosterol-glucoside from *Indigofera zollingeriana* Miq

**Tuong Kha Vo [1+], Qui Thanh Hoai Ta [2+], Quang Truyen Chu [3], Thuy Trang Nguyen[4], and Van Giau Vo [5,6,*]**

[1] Vietnam Sports Hospital, Ministry of Culture, Sports and Tourism, Hanoi 100000, Vietnam
[2] Institute of Research and Development, Duy Tan University, Danang 550000, Vietnam
[3] Institute of Natural Products Chemistry, Vietnam Academy of Science and Technology, Hanoi 100000, Vietnam
[4] Faculty of Pharmacy, Ho Chi Minh City University of Technology (HUTECH), Ho Chi Minh City 700000, Vietnam
[5] Bionanotechnology Research Group, Ton Duc Thang University, Ho Chi Minh City 700000, Vietnam.
[6] Faculty of Pharmacy, Ton Duc Thang University, Ho Chi Minh City 700000, Vietnam
+ These authors contributed equally to this work.
* Correspondence: vovangiau@tdtu.edu.vn

# Supplementary material

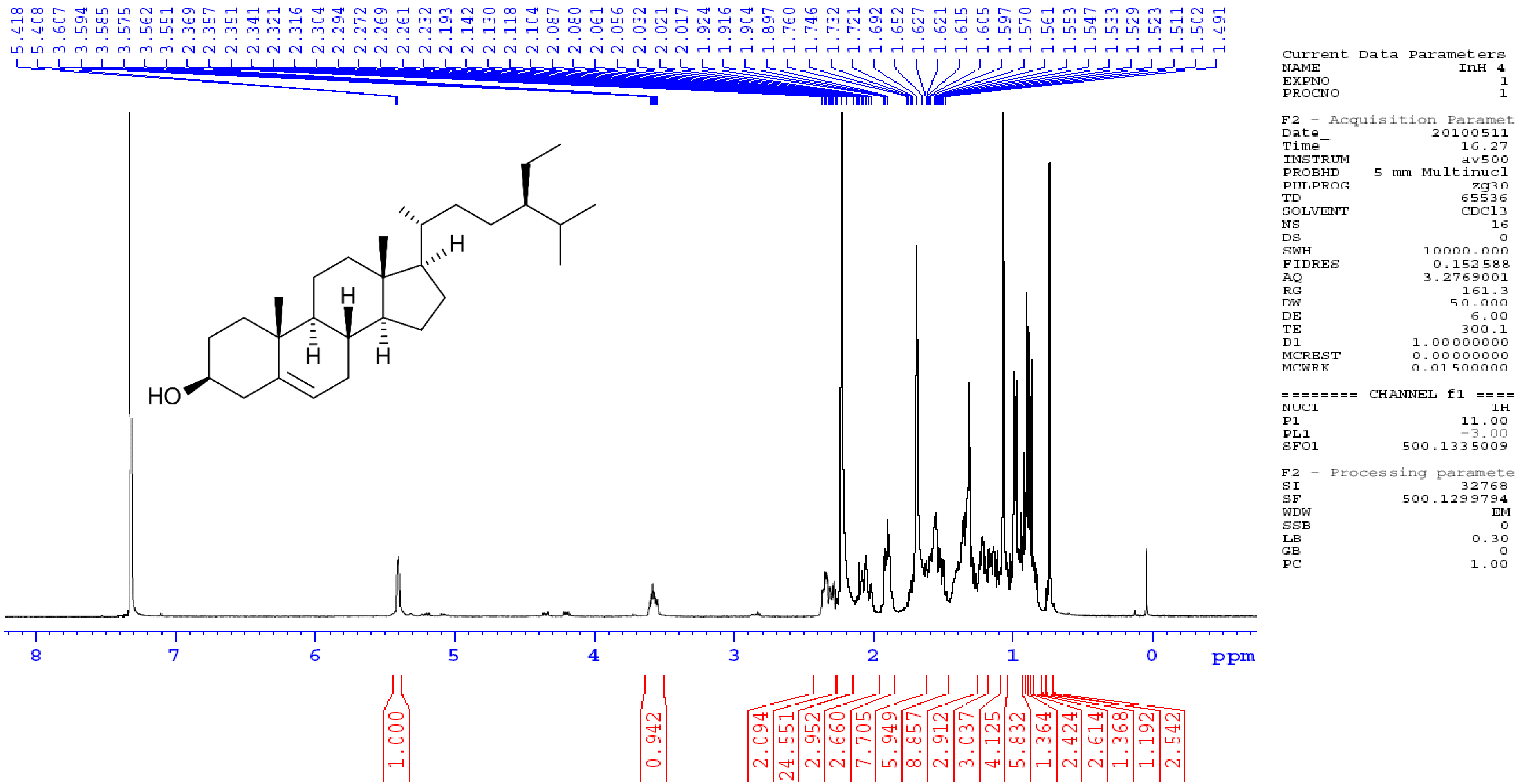

**Figure S1.** $^{13}$C-NMR spectra of β-sitosterol

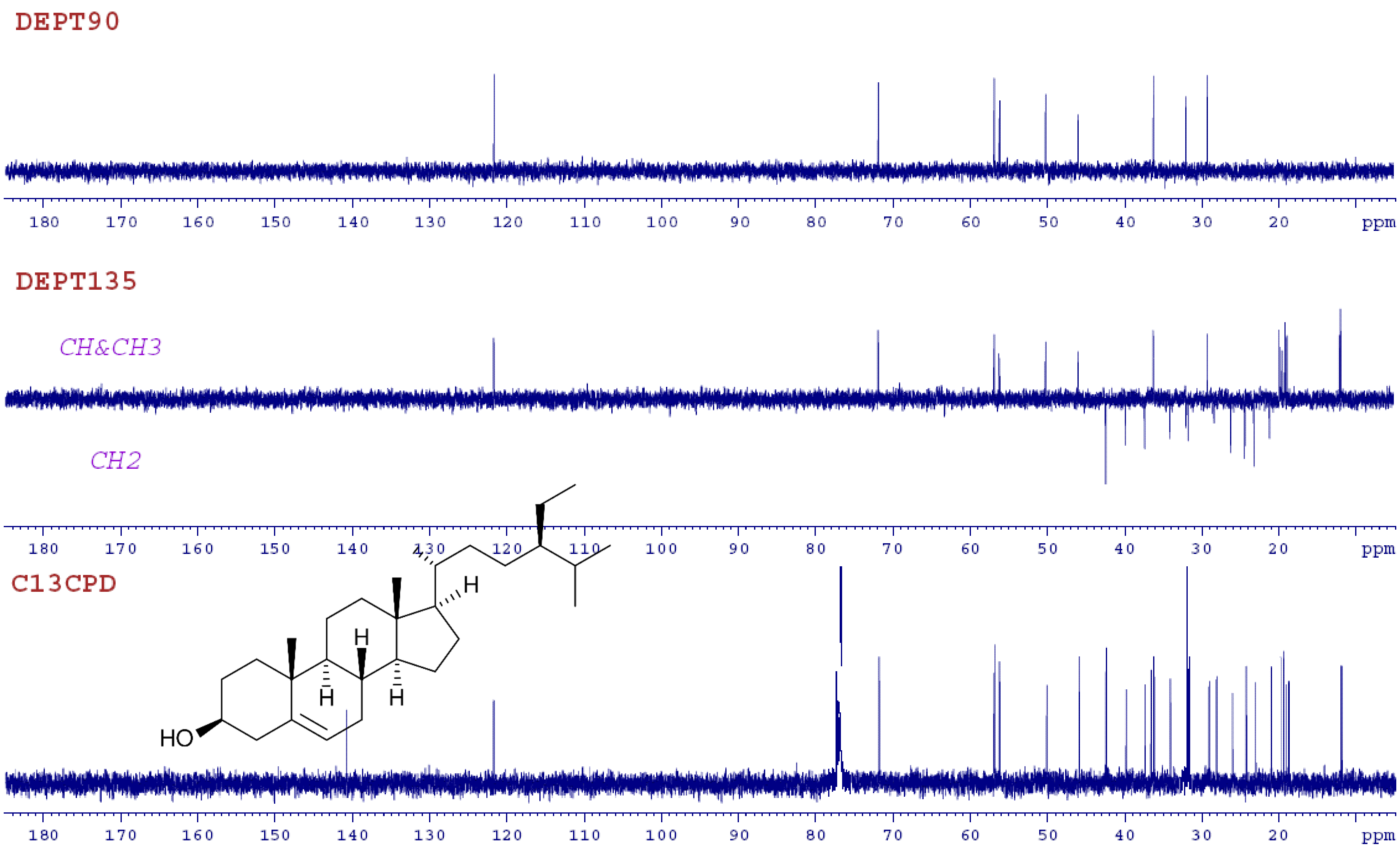


**Figure S2.** $^{13}$C-DEPT 90, 135, and CPD spectra of β-sitosterol

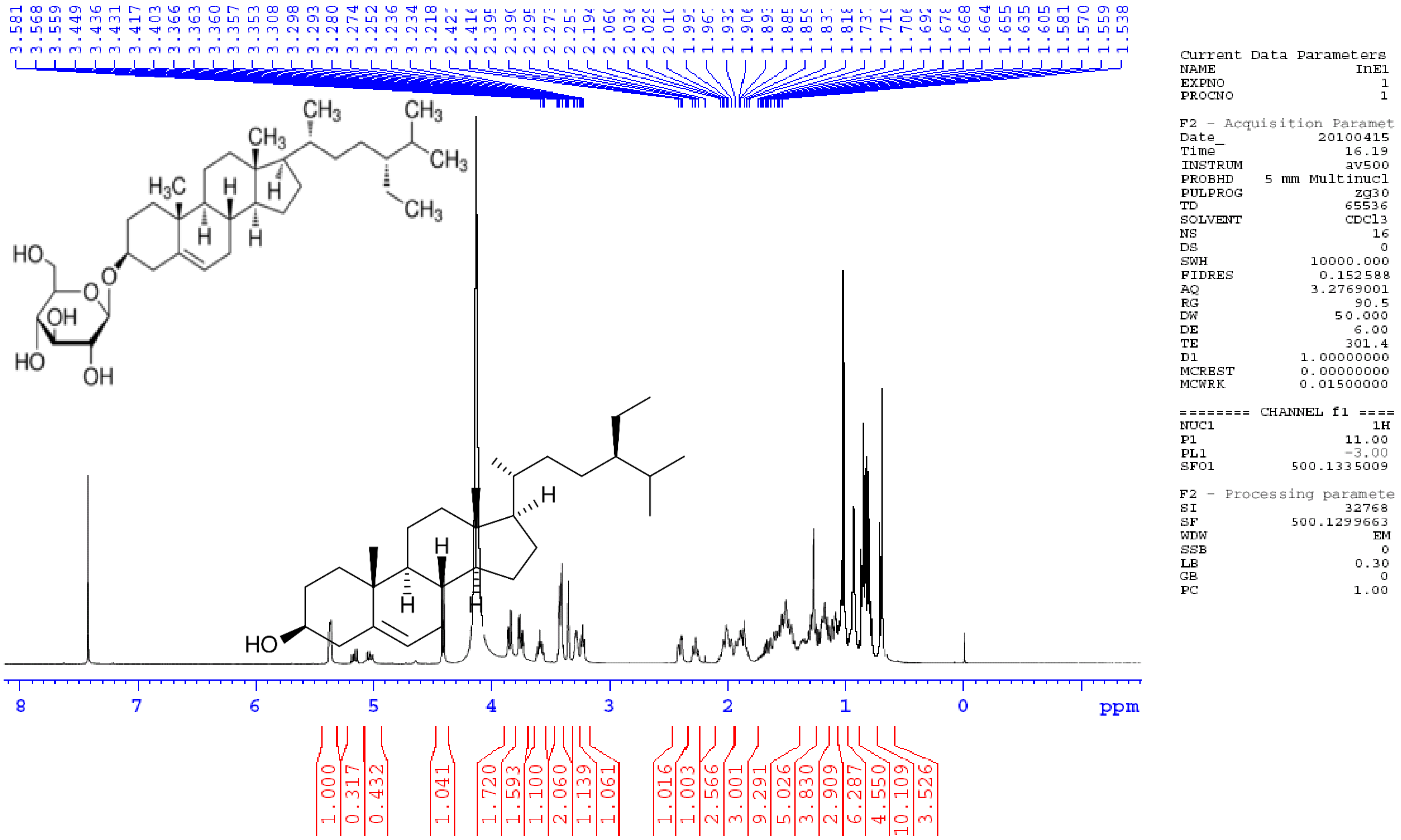


**Figure S3.** $^{1}$H-NMR of β-Sitosterol-glucoside

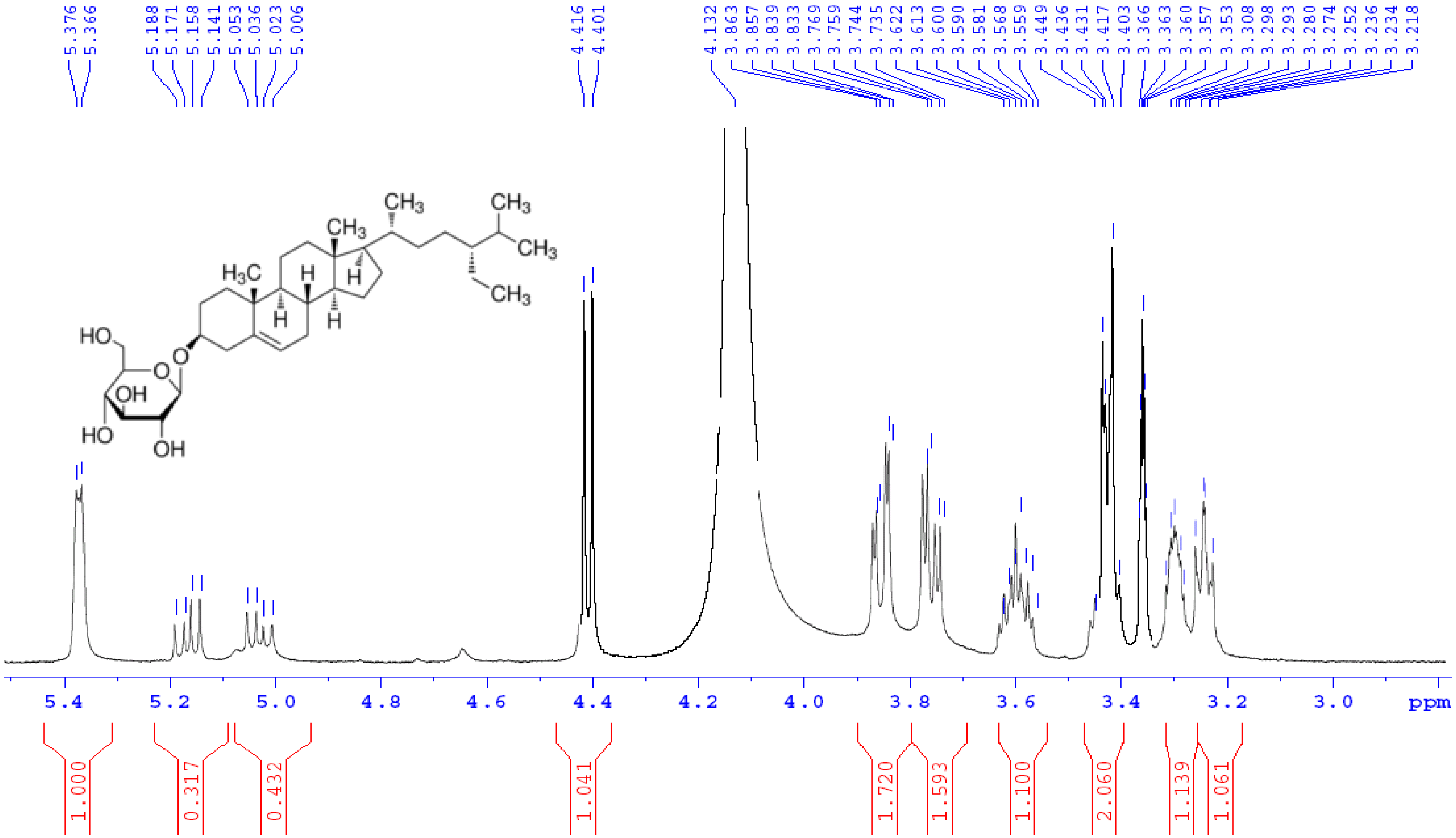


**Figure S4.** $^{1}$H-NMR of β-Sitosterol-glucoside

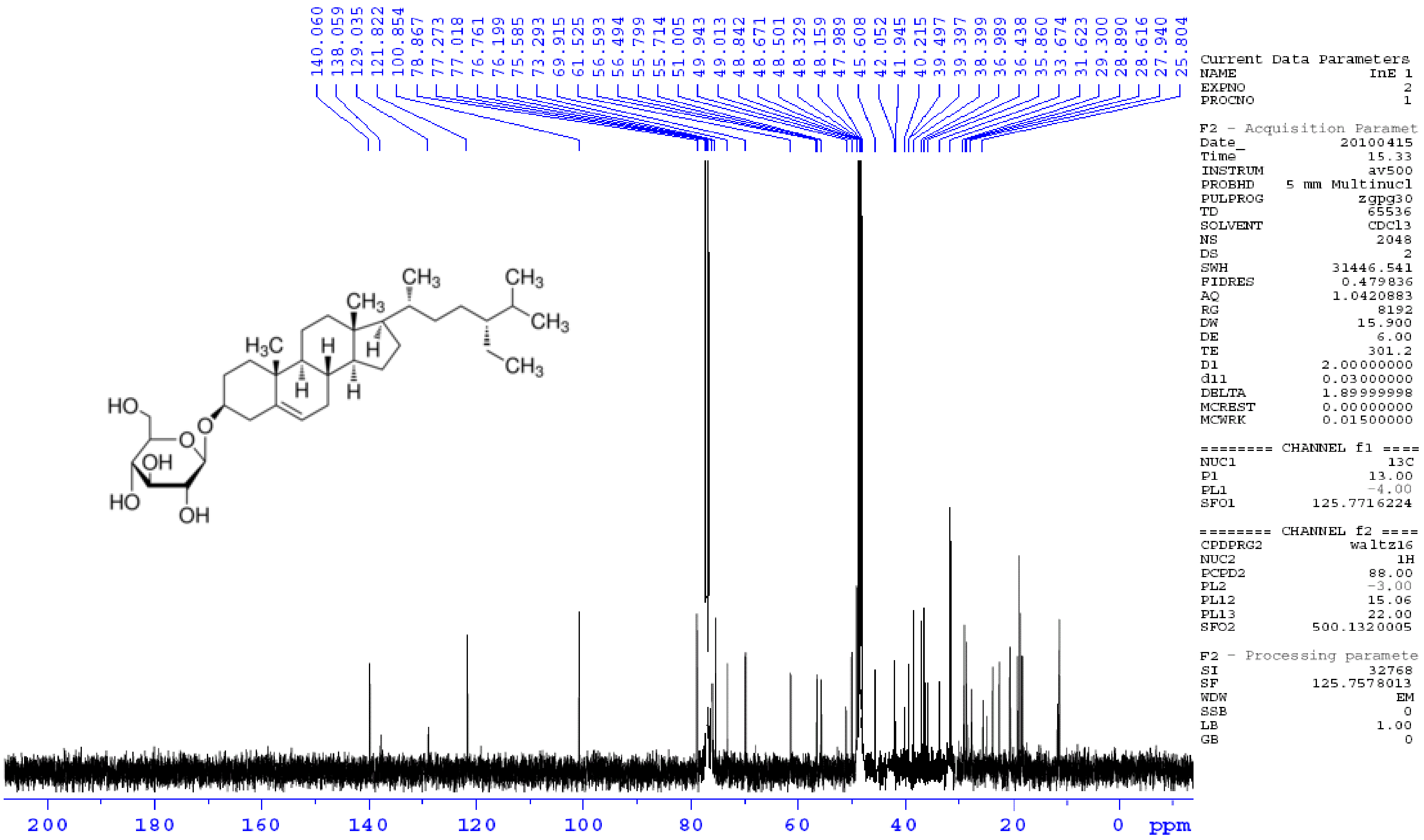

**Figure S5.** $^{13}$C-NMR of β-Sitosterol-glucoside

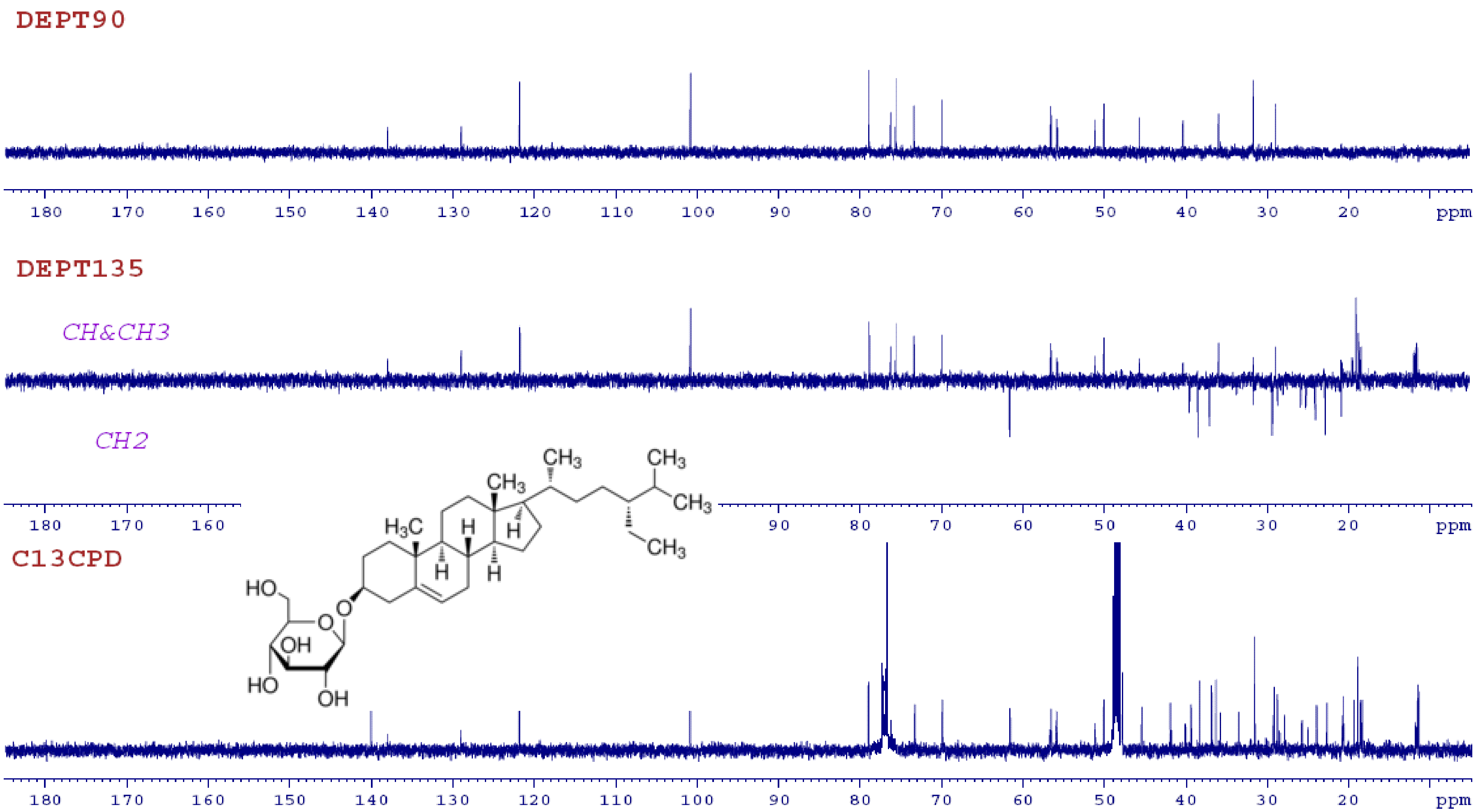


**Figure S6.** $^{13}$C-DEPT 90, 135 and CPD spectra of β-Sitosterol-glucoside

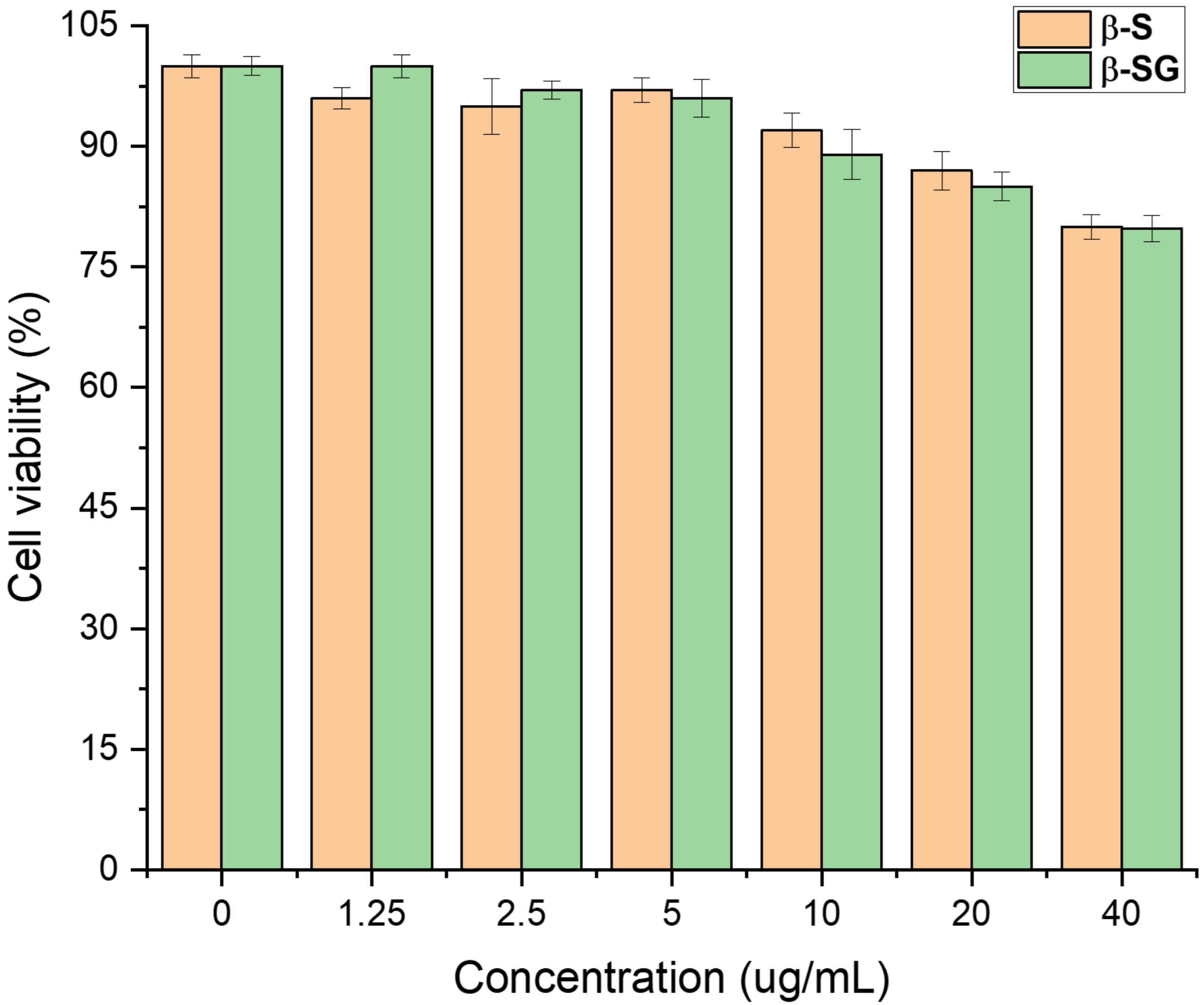


**Figure S7.** Cytotoxicity of the compounds on normal human primary fibroblast (PF) cell at difference concentrations for 48 h

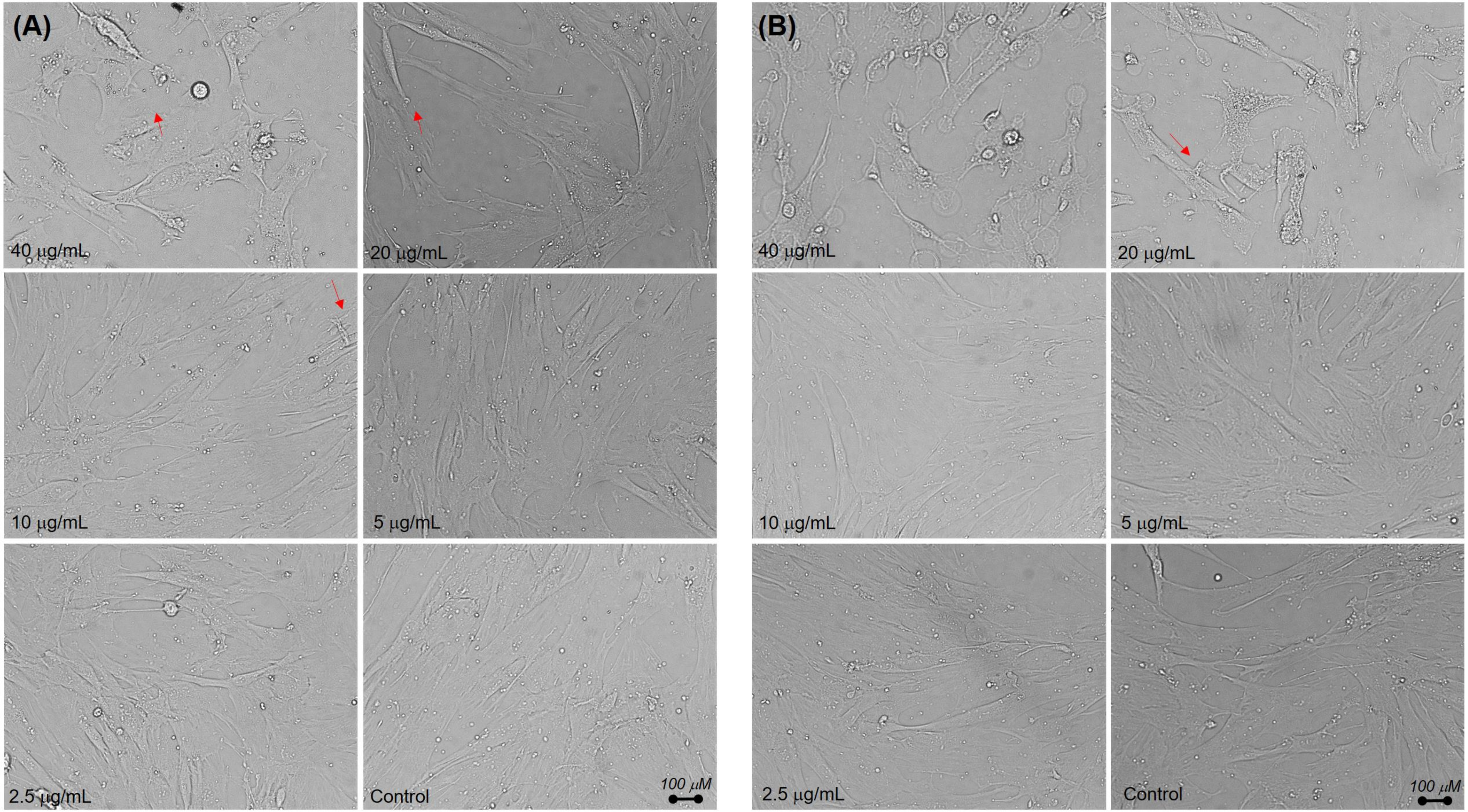


**Figure S8.** Morphological alterations of cells caused by β-S (**A**) and β-SG (**B**) on primary fibroblast (PF) cells at difference concentrations for 48 h
